# Multiphase NiCoFe-Based LDH for Electrocatalytic Sulfion Oxidation Reaction Assisting Efficient Hydrogen Production

**DOI:** 10.3390/ma18143377

**Published:** 2025-07-18

**Authors:** Zengren Liang, Yong Nian, Hao Du, Peng Li, Mei Wang, Guanshui Ma

**Affiliations:** 1 School of Materials Science and Engineering, North University of China, Taiyuan 030051, China; 19935641949@163.com (Z.L.); ny200114ny@gmail.com (Y.N.); 2 School of Energy and Power Engineering, North University of China, Taiyuan 030051, China; 18649390512@163.com (H.D.); lipeng.epe@nuc.edu.cn (P.L.); 3 State Key Laboratory of Advanced Marine Materials, Ningbo Institute of Materials Technology and Engineering, Chinese Academy of Sciences, Ningbo 315201, China; 4 Zhejiang Key Laboratory of Extreme Environmental Material Surfaces and Interfaces, Ningbo Institute of Materials Technology and Engineering, Chinese Academy of Sciences, Ningbo 315201, China

**Keywords:** multiphase NiCoFe-LDH, micron flower structure, sulfion oxidation reaction, hydrogen production, low energy consumption

## Abstract

Sulfion oxidation reaction (SOR) has great potential in replacing oxygen evolution reaction (OER) and boosting highly efficient hydrogen evolution. The development of highly active and stable SOR electrocatalysts is crucial for assisting hydrogen production with low energy consumption. In this work, multiphase NiCoFe-based layered double hydroxide (namely NiCoFe-LDH) has been synthesized via a facile seed-assisted heterogeneous nucleation method. Benefiting from its unique microsized hydrangea-like structure and synergistic active phases, the catalyst delivers substantial catalytic interfaces and reactive centers for SOR. Consequently, NiCoFe-LDH electrode achieves a remarkably low potential of 0.381 V at 10 mA cm^−2^ in 1 M KOH + 0.1 M Na_2_S, representing a significant reduction of 0.98 V compared to conventional OER. Notably, under harsh industrial conditions (6 M KOH + 0.1 M Na_2_S, 80 °C), the electrolysis system based on NiCoFe-LDH||NF pair exhibits a cell potential of only 0.71 V at 100 mA cm^−2^, which shows a greater decreasing amplitude of 1.05 V compared with that of traditional OER/HER systems. Meanwhile, the NiCoFe-LDH||NF couple could maintain operational stability for 100 h without obvious potential fluctuation, as well as possessing a lower energy consumption of 1.42 kWh m^−3^ H_2_. Multiphase eletrocatalysis for SOR could indeed produce hydrogen with low-energy consumption.

## 1. Introduction

Utilizing renewable energy sources (such as electricity converted from solar and wind power) to produce hydrogen via water electrolysis [1,2,3], as a substitute for traditional fossil fuels [4,5,6], represents an effective pathway to address both fossil fuel depletion and global climate change [7,8]. However, in traditional water electrolysis, the anodic oxygen evolution reaction (OER: 4OH^−^ → O_2_ + 2H_2_O + 4e^−^, 1.23 V vs. RHE), involving the breaking of O-H bonds and the formation of O=O bonds, requires the transfer of four electrons, which renders the reaction thermodynamically sluggish and results in elevated cell voltage and excessive energy consumption [9,10]. Currently, an extremely promising and efficient hydrogen production strategy involves introducing additional electrolytes into the electrolyte solution. The anodic oxidation reaction using these additional electrolytes is utilized to replace the slow OER. The oxidation potentials of these additional electrolytes are much lower than that of the traditional OER, for example, urea oxidation reaction (UOR, 0.37 V vs. RHE) [11,12], glucose oxidation reaction (GOR, 0.05 V vs. RHE) [13,14], methanol oxidation reaction (MOR, 0.016 V vs. RHE) [15,16], hydrazine oxidation reaction (HzOR, −0.33 V vs. RHE), [17,18] etc., which can significantly reduce the overpotential of the anode reaction, thereby effectively lowering the overall electrolysis voltage of the electrolytic system. This means that under the same electrolysis voltage, a higher current density and hydrogen production rate can be achieved. It is particularly noteworthy that the sulfion oxidation reaction (SOR, S^2−^ → S + 2e^−^, −0.48 V vs. RHE) involves only two electron transfers during the reaction process, refs. [9,19,20] resulting in a faster reaction kinetics and a lower oxidation potential compared to the aforementioned organic compounds (Table S1). This means that SOR has a more significant advantage in reducing the overpotential of the anode reaction. Therefore, if SOR is coupled with the cathode’s HER, even when operating with a significantly reduced total voltage, the electrolytic system can still maintain efficient HER at the cathode, thereby achieving the goal of reducing energy consumption in hydrogen production.

Consequently, designing a high-performance SOR catalyst is critical. It is noteworthy that transition metal (e.g., Ni, Co, Fe)-based layered double hydroxides (LDHs) demonstrate significant potential in the field of electrocatalytic SOR, owing to their simple synthesis process, abundant active sites, excellent electron transfer properties, and high specific surface area [21,22,23]. For example, the NiFe-LDH/FeNi_2_S_4_/IF constructed by Ai’s team exhibits high mass-transfer efficiency in SOR, benefiting from the superhydrophilic surface and high specific surface area provided by the NiFe-LDH phase [24]. Jiang’s research group reported NiFe-LDH/Cu-IF, where the unique structure of the NiFe-LDH phase—characterized by interconnected porous ultrathin nanosheets with a large specific surface area—significantly contributes to the enhancement of electrocatalytic SOR activity [25].

Inspired by the above, through facile seed-assisted heterogeneous nucleation method [26], this work synthesizes a NiCoFe-LDH catalyst, featuring a unique hydrangea-like microstructure and multiple synergistic active phases (NiCoFe-based (oxy)hydroxides) that endow it with an exceptionally large specific surface area rich in catalytic interfaces and reaction centers, thus delivering exceptional catalytic performance for the SOR. In 1 M KOH and 0.1 M Na_2_S, the NiCoFe-LDH catalyst required an electrolysis potential of only 0.381 V (vs. RHE) at 10 mA cm^−2^, representing a reduction of 0.98 V compared to traditional OER under identical conditions. Long-term stability testing reveals that NiCoFe-LDH catalyst maintains stable operation for 100 h with minimal potential fluctuation. Under industrial operating conditions (6 M KOH + 0.1 M Na_2_S, 80 °C), an SOR/HER electrolysis system based on the NiCoFe-LDH||NF pair requires only 0.71 V to sustain a current density of 100 mA cm^−2^, representing a reduction of 1.05 V compared to conventional OER/HER system. Additionally, the energy consumption for H_2_ production in this system has been reduced to 1.42 kWh m^−3^ H_2_, which is far below the theoretical minimum energy consumption of OER/HER system (2.94 kWh m^−3^ H_2_) [9]. From a theoretical perspective, the adsorptions of S onto Fe_2_O_3_, CoOOH and Ni(OH)_2_ are all spontaneous, and the corresponding intensities are as follows: CoOOH > Ni(OH)_2_ > Fe_2_O_3_. All the above signifies that the SOR/HER electrolysis system based on NiCoFe-LDH||NF pair can achieve the goal of the energy-efficient production of green hydrogen.

## 2. Experimental Section

### 2.1. Materials Preparation

**Scheme 1:**
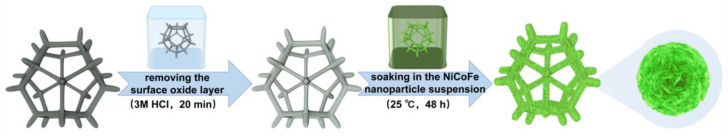
Synthesis process of NiCoFe-LDH catalyst.

The NiCoFe-LDH catalyst was grown in situ on nickel foam (NF, ≥99.99%, thickness of 2  mm, Sinero, Suzhou, China) via the seed-assisted heterogeneous nucleation method (Scheme 1) [26]. Firstly, the nickel foam (5 × 5 cm^2^) was ultrasonically cleaned in acetone and anhydrous ethanol to remove surface oils and other impurities. Secondly, it was immersed in 3 M dilute hydrochloric acid for 20 min to remove the surface oxide layer, then rinsed with deionized water and dried for later use. Next, 14.4 g of nickel (II) nitrate hexahydrate (Ni(NO_3_)_2_·6H_2_O, ≥99.0%, Aladdin Industrial, Shanghai, China) and 3.6 g of cobalt (II) nitrate hexahydrate (Co(NO_3_)_2_·6H_2_O, ≥99.0%, Aladdin Industrial, Shanghai, China) were dissolved in 240 mL of isopropanol to form a mixed solution. Separately, 1.95 g of iron (II) sulfate heptahydrate (FeSO_4_·7H_2_O, ≥99.0%, Aladdin Industrial, Shanghai, China) was dissolved in 80 mL of deionized water. Subsequently, these two solutions were combined and subjected to continuous vigorous stirring for approximately 10 min, followed by natural sedimentation for 5 min to form a stable suspension of NiCoFe nanoparticle seeds. Previous reports indicate that nanoparticles in the suspension facilitate the subsequent in situ growth of the catalyst [26]. Following this, the pretreated NF was immersed in the NiCoFe nanoparticle seed suspension at 25 °C for 48 h. Afterwards, it was rinsed with deionized water and dried in a vacuum oven at 25 °C for 12 h, resulting in the final NiCoFe-LDH catalyst.

### 2.2. Physicochemical Characterization

The phase composition and crystal structure information of the NiCoFe-LDH catalyst powder were characterized using X-ray diffraction (XRD, DX-2700BH, Haoyuan Instrument Co., Ltd., Dandong, China) with Cu Kα radiation (λ = 0.154 nm) operating at 40 kV and 30 mA. The concentration of vacancy defects was evaluated by electron paramagnetic resonance (EPR, JES-FA200, JEOL, Tokyo, Japan). The microstructure of the material was imaged using scanning electron microscopy (SEM, JSM-7900F, JEOL, Tokyo, Japan).

### 2.3. Electrochemical Measurements

The as-prepared NiCoFe-LDH is a self-supporting electrode that can be directly used for testing and does not need a binder, while the control RuO_2_ (99.9%, Macklin reagent, Shanghai, China) electrode should be prepared as follows: 4 mg RuO_2_ powder and 1 mg carbon black (Vulcan XC-72, Cabot, Boston, MA, USA) were dispersed in 300 μL isopropanol, followed by adding 100 μL Nafion solution (5 wt%, DuPont, Nogales, AZ, USA). The mixture was ultrasonicated to form a homogeneous RuO_2_ catalyst ink. Subsequently, 50 μL of the RuO_2_ catalyst ink was loaded onto a 1 cm^2^ nickel foam (NF) substrate, resulting in a mass loading of approximately 0.5 mg cm^−2^.

For the half-reaction electrochemical tests, measurements were performed at room temperature using a three-electrode system connected to an electrochemical workstation (CS2350H, CorrTest, Wuhan, China). The catalyst electrode served as the working electrode (WE), a platinum sheet as the counter electrode (CE), and an Hg/HgO electrode as the reference electrode (RE). All half-reaction potentials were converted to the reversible hydrogen electrode (RHE) scale according to the following equation:

E_RHE_ = E_Hg/HgO_ + 0.098 + 0.059 × pH (1)

The potentiodynamic scan test, one of the half-reaction tests, was performed at a scanning rate of 0.05 mV/s to evaluate the corrosive effect of electrolytes containing different concentrations of Sodium sulfide hexahydrate (Na_2_S·6H_2_O, ≥98.0%, Aladdin Industrial, Shanghai, China) on the NiCoFe-LDH electrode. In addition, half-reaction tests also included measurements of alkaline OER and SOR in 1.0 M KOH (≥85.0%, Damao Chemical Reagent, Tianjin, China) with and without Na_2_S present (i.e., in 1.0 M KOH and 1.0 M KOH + 0.1 M Na_2_S). OER and SOR activities were evaluated by linear-sweep voltammetry (LSV) at a scan rate of 5 mV s^−1^. All test results were recorded without iR compensation. Tafel slopes were extracted from the LSV data according to the following equation:

η = b × log|j| + a(2)

where η, b, j, and a represent the overpotential, Tafel slope, current density, and Tafel constant, respectively. Electrochemical impedance spectroscopy (EIS) measurements were conducted in the frequency range from 10^5^ Hz to 0.01 Hz at a constant potential of 1.4 V vs. RHE (in 1.0 M KOH). SOR stability was evaluated through chronopotentiometry (CP) at a constant current density of 10 mA cm^−2^. The ECSA reflects intrinsic catalyst properties and can be calculated using the double-layer capacitance (C_dl_) values of catalyst according to the following equation:

ECSA = C_dl_/C_s_ = Cdl/(40 μ^−2^per^2^_ECSA_) (3)

Among these, the Cdl value was determined by conducting cyclic voltammetry (CV) measurements within the non-Faradaic potential region (OCP ± 0.05 V vs. RHE), at various scan rates ranging from 5 to 100 mV s^−1^. The slope was then extracted from the plot of |ja − jc|/2 versus scan rate. C_s_ is typically chosen as 40 µF cm^−2^. Furthermore, SOR-coupled HER water electrolysis was tested in an anion exchange membrane (AEM) electrolyzer. The electrolyzer employed NiCoFe-LDH as the anode and nickel foam (NF) as the cathode, both with an electrode area of 2 cm × 2 cm. The electrolyte was 1.0 M KOH + 0.1 M Na_2_S at 80 °C. Polarization curves were recorded at a scan rate of 5 mV s^−1^, and stability was measured at a current density of 100 mA cm^−2^.

### 2.4. Theoretical Calculation Method

Theoretical calculations were performed using density functional theory (DFT) as implemented in the Vienna ab initio simulation package (VASP 6.3.0). The Perdew–Burke–Ernzerhof (PBE) functional was employed. To address the challenges of highly localized and strongly correlated electronic systems, a DFT + U correction was applied. The Hubbard U values were set to 3.4 eV for Ni, 3.4 eV for Co, and 3.3 eV for Fe. To prevent interactions between periodic images of the slab, a vacuum layer thickness of 15 Å was introduced, with 7.5 Å above and 7.5 Å below the catalyst slab. Additional computational parameters included a plane-wave cutoff energy of 400 eV, a self-consistent field (SCF) convergence threshold of 10^−5^ eV, an energy convergence criterion of 0.03 eV Å^−1^ for ionic relaxation, and a k-point mesh of 5 × 5 × 1.

## 3. Results and Discussion

### 3.1. Microstructure Analysis

**Figure 1:**
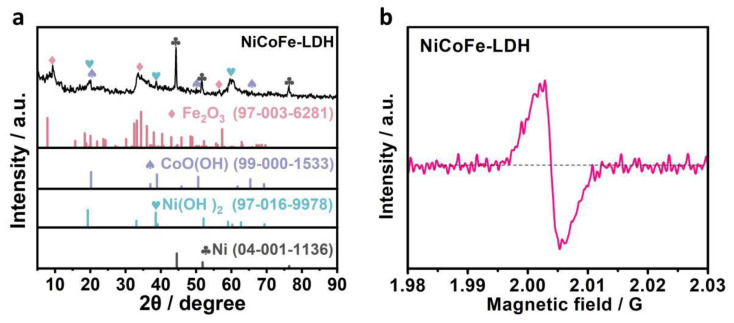
(**a**) XRD pattern and (**b**) EPR spectra of NiCoFe-LDH.

To avoid the characteristic peaks of the NiCoFe-LDH catalyst being masked by those of the nickel foam (NF) substrate, the NiCoFe-LDH powder was scraped off the NF surface. Phase analysis of the catalyst powder was then performed using X-ray diffraction (XRD). The phase analysis revealed that NiCoFe-LDH was not a single phase, but rather composed of Ni(OH)_2_ (PDF#97-016-9978), CoO(OH) (PDF#99-000-1533), Fe_2_O_3_ (PDF#97-003-6281) and Ni (PDF#04-001-1136) phases (Figure 1a). Thereinto, the detection of Ni was due to the inadvertent scraping of the surface layer of the NF substrate during the removal of the NiCoFe-LDH powder, which subsequently became mixed with the catalyst powder. The post-generated multiphase (oxy)hydroxides contain numerous oxygen vacancy defects (Figure 1b), which may serve as catalytic active sites to promote the adsorption and conversion of reactants and thereby enhancing catalytic performance [27,28].

**Figure 2:**
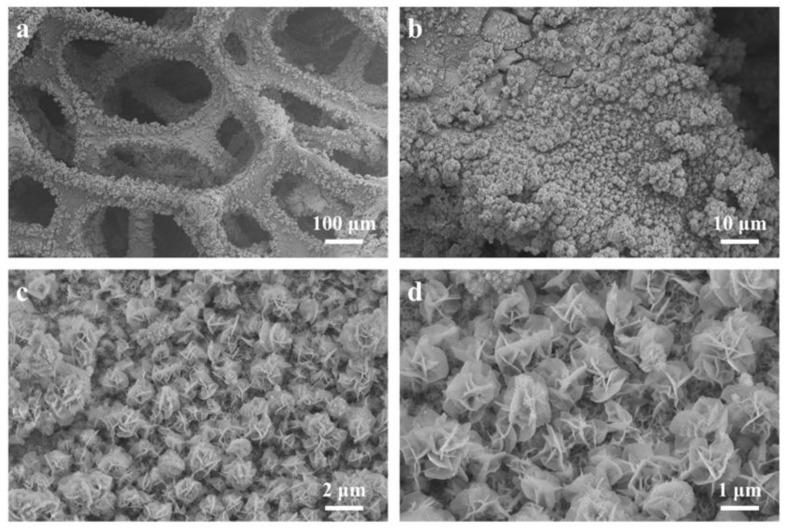
(**a**–**d**) Low- and high-magnification SEM images of NiCoFe-LDH.

As shown in the scanning electron microscopy (SEM) images (Figure 2), the nickel foam (NF) surface is uniformly covered with dense flake-like clusters of NiCoFe-LDH catalyst. On the upper layer of these clusters, the surface morphology of catalyst exhibits a hydrangea-like shape at the micrometer scale. The formation of this unique surface morphology can be attributed to the natural crystallization of the NiCoFe-LDH catalyst on the surface of NF, which significantly increases the specific surface area of catalyst, thereby providing more active sites for catalytic reactions and enhancing mass-transfer efficiency.

### 3.2. Electrocatalytic Activity

**Figure 3:**
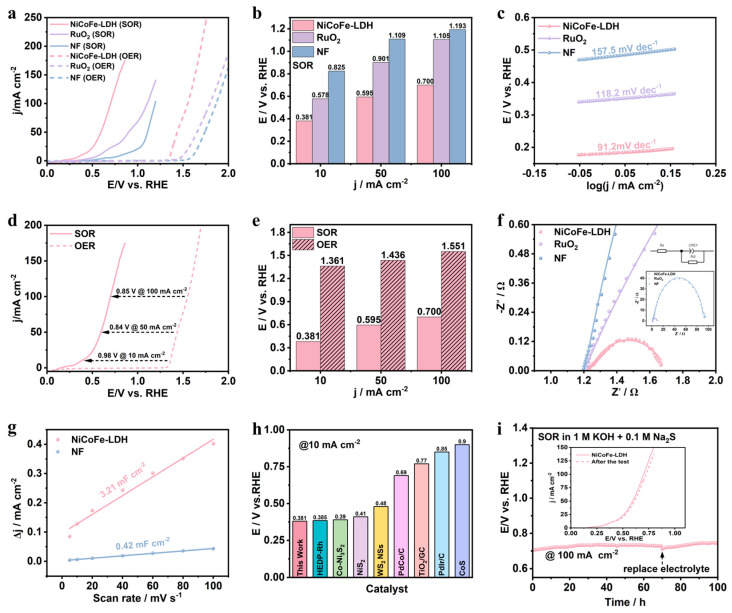
(**a**) LSV curves in 1 M KOH and 1 M KOH + 0.1 M Na_2_S; (**b**) comparison of SOR performances at 10, 50 and 100 mA cm^−2^; (**c**) Tafel plots; (**d**,**e**) comparison of OER and SOR of NiCoFe-LDH; (**f**) Nyquist plots; (**g**) Cdl values; (**h**) SOR performance contrast at 10 mA cm^−2^; (**i**) CP profile at 100 mA cm^−2^—the inset shows LSV curves before and after stability test.

This work systematically evaluated the electrocatalytic activity of the synthesized NiCoFe-LDH catalyst toward the SOR. It should be noted that S^2−^ has a corrosive effect on the catalyst [29,30]. In this work, through potentiodynamic scanning tests, the potentiodynamic polarization curve of the NiCoFe-LDH electrode in an electrolyte solution of 1 M KOH + (0.1–0.5) M Na_2_S were obtained (Figure S1), and corrosion potential (E_corr_) and corrosion current density (I_corr_) were analyzed (Table S2). Among them, the electrolyte containing 1 M KOH + 0.1 M Na_2_S exhibited the highest E_corr_ (−0.649 V) and the lowest I_corr_ (1.53 × 10^−3^ A cm^−2^), indicating that it had the least corrosive effect on the NiCoFe-LDH electrode [31]. As a result, 0.1 M Na_2_S was determined as the typical concentration to be used in the subsequent SOR performance tests. LSV curves in 1 M KOH and in 1 M KOH with added 0.1 M Na_2_S demonstrate that the NiCoFe-LDH-catalyzed reaction achieves a higher current density at the same potential, indicating significantly enhanced OER and SOR catalytic performance compared to commercial nickel foam (NF) and RuO_2_ (Figure 3a). At current densities of 10 mA cm^−2^, 50 mA cm^−2^, and 100 mA cm^−2^, the SOR driving potential for NiCoFe-LDH is only 0.381 V, 0.595 V, and 0.7 V vs. RHE, respectively. These values are markedly lower than those for NF and RuO_2_ (Figure 3b). Tafel slope is a key kinetic parameter for evaluating electrochemical catalytic performance and plays a crucial role in assessing catalyst activity. It provides essential insights for studying the kinetics of the SOR. A lower Tafel slope value indicates reaction kinetics under the action of catalyst, attributed to optimized reaction pathway and improved charge-transfer efficiency [32]. The catalytic kinetics of the SOR were systematically studied by deriving and analyzing Tafel slopes based on LSV curves. As shown in Figure 3c, the Tafel slope for NiCoFe-LDH is 91.2 mV dec^−1^, lower than those of commercial nickel foam (NF) (118.2 mV dec^−1^) and RuO_2_ (157.5 mV dec^−1^). This demonstrates the superiority of NiCoFe-LDH in the reaction kinetics of the SOR. To contrast with the conventional OER, the LSV curves of NiCoFe-LDH in 1 M KOH + 0.1 M Na_2_S were compared with those in 1 M KOH without Na_2_S (Figure 3d,e). It can be seen that in the presence of 0.1 M Na_2_S, the potentials at current densities of 10, 50, and 100 mA cm^−2^ are 0.98 V, 0.84 V, and 0.85 V (vs. RHE) lower, respectively, than those required for conventional OER.

To obtain a quantitative analysis of the catalyst kinetics, the equivalent circuit model shown in Figure 3f was used to fit the electrochemical impedance spectroscopy (EIS) data. The fitting results revealed that the NiCoFe-LDH electrode exhibits the smallest semicircle radius and the lowest charge-transfer resistance (R_ct_) of 0.4704 Ω (Table S3), compared to NF and RuO_2_. This indicates that NiCoFe-LDH possesses a faster charge-transfer rate and higher electrical conductivity [19]. To gain deeper insight into the superior SOR catalytic activity of NiCoFe-LDH, the double-layer capacitance (C_dl_) derived from Figure 3g was used to estimate the electrochemically active surface area (ECSA) of catalyst. Cyclic voltammetry (CV) measurements at various scan rates (5–100 mV s^−1^, Figure S2) were conducted within a non-Faradaic potential window (OCP ± 0.05 V vs. RHE) to avoid interference from redox reactions. The C_dl_ values for NF and NiCoFe-LDH, calculated from the linear slope of the plot of current density difference at the open-circuit potential (Δj/2) versus scan rate, were determined to be 0.42 mF cm^−2^ and 3.21 mF cm^−2^, respectively. The corresponding ECSA values, calculated according to Equation (3), are 10.5 cm^−2^ and 80.25 cm^−2^, respectively. It is evident that the unique structure of hydrangea-like microspheres stacked on flake-like clusters endows NiCoFe-LDH with a significantly larger catalytic surface area and more active sites compared to bare NF. This explains its outstanding SOR catalytic performance. Compared with other reported catalysts, NiCoFe-LDH achieves excellent catalytic performance at lower concentrations of Na_2_S, whereas other catalysts rely on higher Na_2_S concentrations to enhance the reaction rate and reduce the required potential (Figure 3h, Table S4) [33,34,35,36,37,38,39,40]. This indicates that the SOR catalytic activity of NiCoFe-LDH surpasses that of most previously reported catalysts. Furthermore, in this work, the pH of the electrolyte without Na_2_S addition, with Na_2_S addition, and after the SOR were measured using the pH meter. The results showed that there were no significant differences among these three cases (Figure S3), thus eliminating the interference of pH changes on the performance evaluation of SOR.

**Figure 4:**
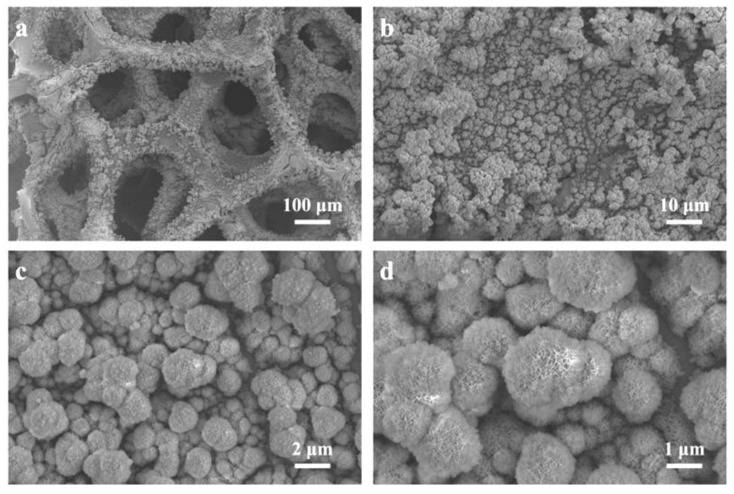
(**a**–**d**) SEM images of NiCoFe-LDH after 100 h stability test in 1 M KOH + 0.1 M Na_2_S electrolyte.

To assess the mechanical and chemical stability of NiCoFe-LDH, a chronopotentiometry (CP) measurement was conducted at a constant current density of 100 mA cm^−2^ for 100 h (Figure 3i). The results demonstrate outstanding stability, with a negligible potential fluctuation of only 40 mV during prolonged operation. After stability testing, the NiCoFe-LDH catalyst grown on the surface of NF underwent in situ transformation into a colony-like structure firmly anchored to the substrate (Figure 4). This robust structure ensures the continuous provision of a large active surface area during catalytic reactions, facilitating efficient mass transport, which is proved by the extremely minor potential changes (23 mV at 100 mA cm^−2^) before and after the stability test (inset of Figure 3i).

**Figure 5:**
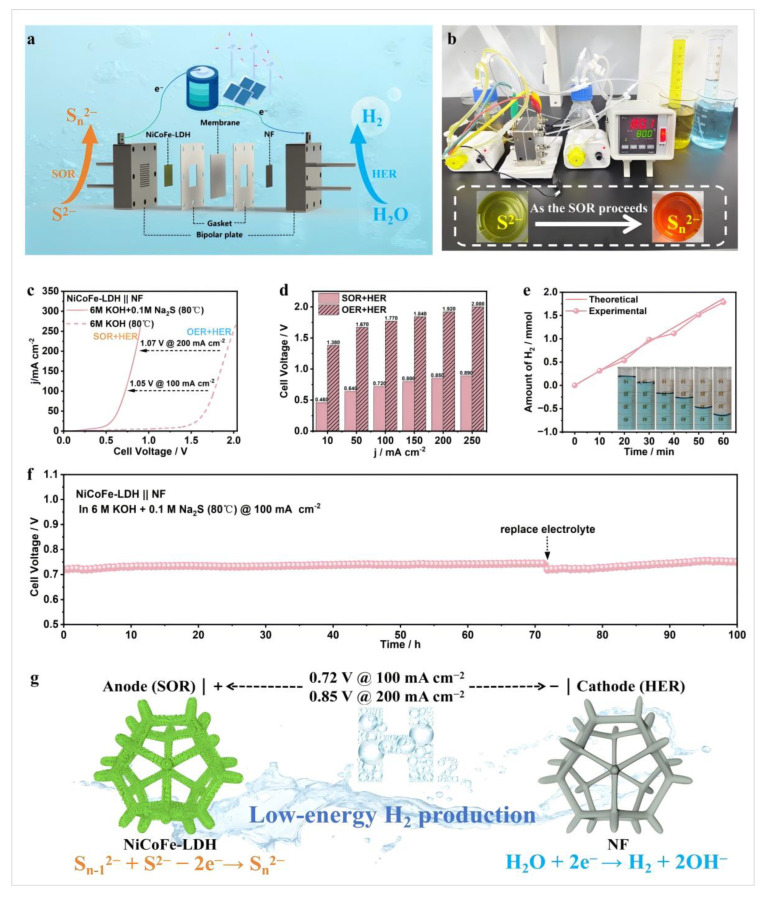
(**a**) A schematic diagram and (**b**) photograph of SOR/HER electrolysis system based on NiCoFe-LDH||NF pair—the inset shows the change in electrolyte color as the SOR progresses; (**c**) LSV curves of SOR/HER and OER/HER electrolysis system; (**d**) a comparison of cell voltages at 10–250 mA cm^−2^; (**e**) the amount of the theoretically calculated and experimentally measured H_2_ production—the inset is an illustration of H_2_ yields at 100 mA for 60 min; (**f**) CP profiles at 100 mA cm^−2^; (**g**) a mechanistic diagram for the effect of SOR on HER.

Leveraging the outstanding SOR catalytic activity of the NiCoFe-LDH catalyst, an AEM electrolyzer was employed to construct an SOR-coupled HER electrolysis system, as illustrated in Figure 5a,b. In this system, NiCoFe-LDH and NF served as the anode and cathode, respectively (both with an electrode area of 2 cm × 2 cm), to evaluate the performance of SOR-assisted water electrolysis for hydrogen production. The testing of this system was conducted under simulated industrial operating conditions (6 M KOH + 0.1 M Na_2_S, 80 °C). The S^2−^ is oxidized to elemental sulfur (S) at the anode of the AEM electrolyzer, catalyzed by NiCoFe-LDH. The generated S subsequently reacts with excess S^2−^ in the electrolyte to form soluble polysulfides (S_n_^2−^), thereby achieving S^2−^ degradation, while green hydrogen is simultaneously produced at the cathode (Figure 5a). As shown in the inset of Figure 5b, the anode electrolyte gradually turned yellow, indicating the progressive accumulation of S_n_^2−^ during the SOR process [41,42,43]. Figure 5c presents the LSV curves for the NiCoFe-LDH||NF electrode pair in both the SOR/HER electrolysis system and the conventional OER/HER electrolysis system. It is evident that at the same applied cell voltage, the SOR/HER system achieves significantly higher current densities than the traditional OER/HER system. The cell potentials for the SOR-coupled HER system at current densities of 100 and 200 mA cm^−2^ are only 0.72 V and 0.85 V, respectively, representing reductions of 1.05 V and 1.07 V compared to the conventional water electrolysis system (Figure 5d). Consequently, the corresponding electrical energy consumption is reduced by 59.3% and 55.7%, respectively. This significant energy saving is primarily attributed to the faster kinetics of the anodic SOR compared to OER, highlighting the crucial role of SOR in lowering the electrolysis potential required for water splitting. The above information demonstrates that the NiCoFe-LDH-catalyzed SOR-coupled HER electrolysis system achieves the objective of energy-efficient hydrogen production.

To evaluate the hydrogen production efficiency of this system, electrolysis was conducted at a constant current of 100 mA (current density of 25 mA cm^−2^) for one hour. The hydrogen gas produced was collected and measured via the water-displacement method. The experimentally measured hydrogen yield was nearly identical to the theoretical value (Figure 5e). The Faradaic efficiency (FE) was calculated to be as high as 95.67% based on the ratio of the actual yield to the theoretical yield. The energy consumption for hydrogen production by this electrolysis system, calculated from the electrical energy consumed during electrolysis and the measured actual hydrogen yield, was determined to be 1.42 kWh m^−3^ H_2_ (only for electrolysis, excluding losses such as pumping, gas separation and system heat loss). This is significantly lower than the theoretical minimum energy consumption (2.94 kWh m^−3^ H_2_) for OER/HER electrolysis systems [9]. To verify its feasibility under harsh industrial operating conditions, a durability test was performed at a current density of 100 mA cm^−2^. The NiCoFe-LDH catalyst maintained stable operation for 100 h at 100 mA cm^−2^ with negligible voltage fluctuation (Figure 5f). This indicates its ability to meet the stability requirements for industrial-scale applications, demonstrating its strong potential for industrial-scale deployment. In Figure 5g, the mechanistic diagram of SOR/HER system is illustrated, which clarifies the promoting effect of SOR on the alkaline water electrolysis.

### 3.3. Theoretical Analysis

**Figure 6:**
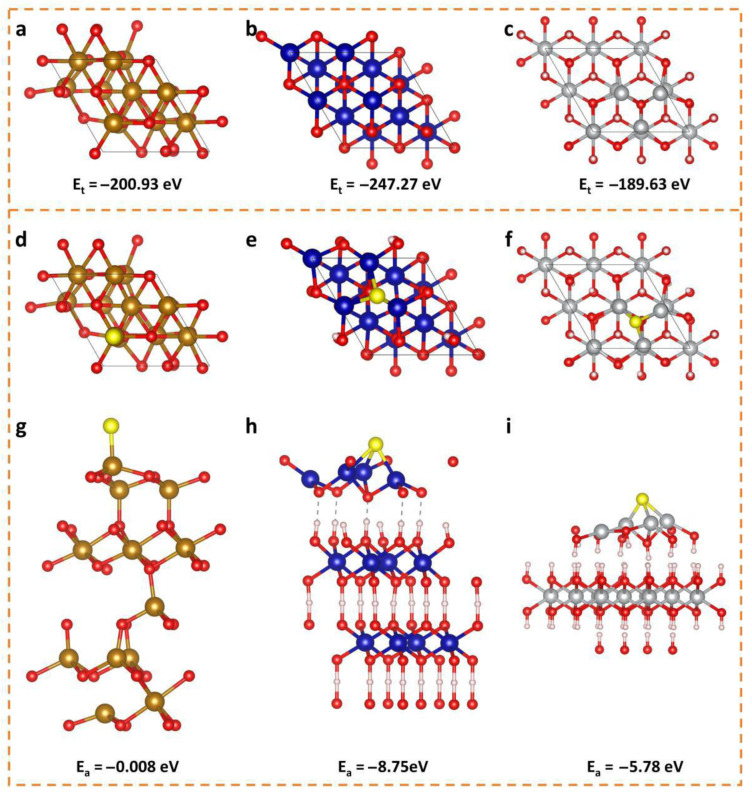
The slab models and corresponding total energies of (**a**) Fe_2_O_3_, (**b**) CoOOH, and (**c**) Ni(OH)_2_. (**d**–**f**) The top view of optimized *S adsorption models. (**g**–**i**) A side view and corresponding adsorption energies of optimized *S adsorption models.

From the above experiments, it can be found that multiphase NiCoFe-LDH mainly consists of Fe_2_O_3_, CoOOH, and Ni(OH)_2_, which may all make contributions to electrocatalytic SOR. From Figure 6a–c, it can be seen that the total energies of the three slab models are different from each other, which could explain the distinct adsorption energies of *S on them. Through accurate calculation, the adsorption energies of S onto Fe_2_O_3_, CoOOH and Ni(OH)_2_ are all negative values, proving that the *S adsorption on them are all exothermic processes and spontaneous. After a comparison of the corresponding *S adsorption intensities, the following rules can be established: CoOOH > Ni(OH)_2_ > Fe_2_O_3_, which attests that CoOOH is more active for SOR than the other two.

## 4. Conclusions

In summary, this study synthesized a multiphase NiCoFe-based LDH (namely NiCoFe-LDH) via a facile seed-assisted heterogeneous nucleation approach. The NiCoFe-LDH possesses a unique micro-sized hydrangea-like morphology with an extremely large specific surface area and synergistically active phases, providing abundant catalytic interfaces and reactive centers for the SOR. Consequently, the NiCoFe-LDH catalytic electrode achieved an ultralow electrolysis potential of merely 0.381 V at 10 mA cm^−2^ in 1 M KOH + 0.1 M Na_2_S electrolyte, significantly reducing the potential by 0.98 V compared to the traditional OER. Under stringent industrial operating conditions (6 M KOH + 0.1 M Na_2_S, 80 °C), the electrolysis potential for the SOR/HER system based on the NiCoFe-LDH||NF electrode pair was reduced to 0.71 V at 100 mA cm^−2^, demonstrating a substantial reduction of 1.05 V compared to the conventional OER/HER system. Furthermore, the NiCoFe-LDH||NF electrode pair maintained stable hydrogen production and S^2−^ oxidation or degradation for 100 h with negligible potential fluctuation, while the energy consumption for hydrogen production was lowered to 1.42 kWh m^−3^ H_2_. From a theoretical perspective, the adsorptions of S onto Fe_2_O_3_, CoOOH, and Ni(OH)_2_ are all spontaneous, and the corresponding intensities are as follows: CoOOH > Ni(OH)_2_ > Fe_2_O_3_. Evidently, the outstanding SOR catalytic performance of NiCoFe-LDH renders it highly promising for achieving industrial-scale energy-efficient production of green hydrogen.

## Data Availability

The original contributions presented in this study are included in the article/Supplementary Materials. Further inquiries can be directed to the corresponding authors.

## Supplementary Materials

The following supporting information can be downloaded at: https://www.mdpi.com/article/10.3390/ma18143377/s1, Figure S1: Potentiodynamic polarization curve of NiCoFe-LDH in 1 M KOH + (0.1–0.5) M Na_2_S; Figure S2: CV curves (OCP ± 0.05 V vs. RHE) of (a) NiCoFe-LDH and (b) NF electrodes under different scan rates (5–100 mV s^−1^); Figure S3: The pH of the electrolyte without Na_2_S addition, with Na_2_S addition, and after the SOR. Table S1: A comparison of the electro-oxidation potentials of the SOR with those of other electrolyte oxidation reactions; Table S2: Corrosion potential (E_corr_) and corrosion current density (I_corr_) of NICoFe-LDH in 1 M KOH + (0.1–0.5) M Na_2_S; Table S3: Electrochemical impedance parameters obtained by fitting the Nyquist plots of NiCoFe-LDH, RuO_2_, and NF to the equivalent circuit mode; Table S4: A comparison of the electrochemical SOR activities of this work with recent outstanding reported electrocatalysts.
