# Supplementary material for: Multiphase NiCoFe-Based LDH for Electrocatalytic Sulfion Oxidation Reaction Assisting Efficient Hydrogen Production
Source: Materials (Basel). 2025 Jul 18;18(14):3377. doi: 10.3390/ma18143377 (PMC12298416; doi:10.3390/ma18143377)
Supplement: Supplementary file 1 [file materials-18-03377-s001.zip › materials-3740234-supplementary.pdf]

Supporting Information

Multiphase NiCoFe-Based LDH For Electrocatalytic Sulfion Oxidation Reaction Assisting Efficient Hydrogen Production

Supplementary Figures

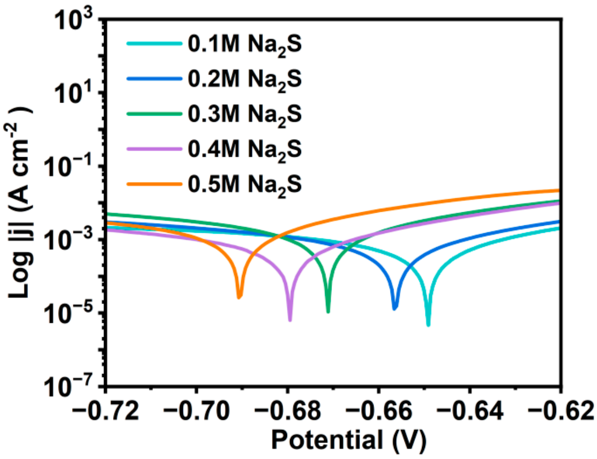

Figure S1. Potentiodynamic polarization curve of NiCoFe-LDH in 1 M KOH + (0.1-0.5) M Na_2S.

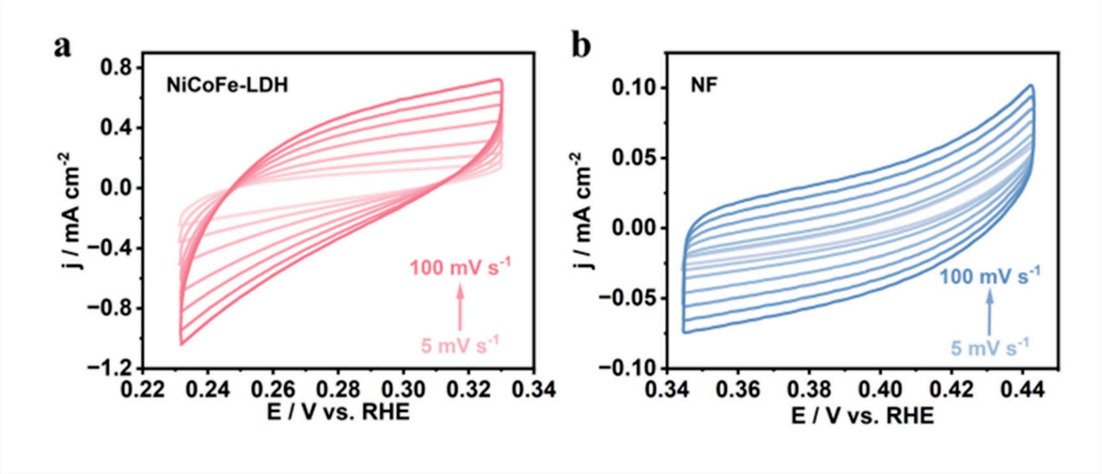

Figure S2. CV curves (OCP±0.05 V vs. RHE) of (a) NiCoFe-LDH and (b) NF electrodes under different scan rates (5-100 mV s^{-1}).

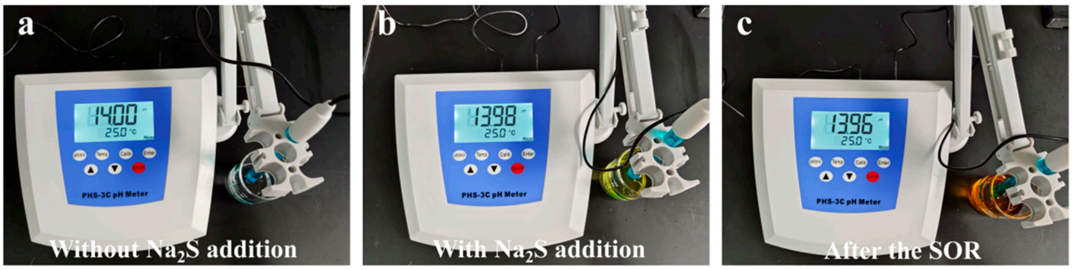

Figure S3. The pH of the electrolyte without Na_2S addition, with Na_2S addition, and after the SOR.

Supplementary Table

Table S1. A comparison of the electro-oxidation potentials of the SOR with those of other electrolyte oxidation reactions.

**Electrolyte**	Reaction	Potential (V vs. RHE)	Reference
**Sulfion**	SOR	-0.48	[1,2]
**Hydrazine**	HzOR	-0.33	[3,4]
**Methanol**	MOR	0.016	[5,6]
**Glucose**	GOR	0.05	[7,8]
**Urea**	UOR	0.37	[9,10]

Table S2. Corrosion potential (E_{corr}) and corrosion current density (I_{corr}) of NICoFe-LDH in 1 M KOH + (0.1-0.5) M Na_2S.

**Concentration of Na_2S / M**	0.1	0.2	0.3	0.4	0.5
**E_{corr} / V**	-0.649	-0.656	-0.671	-0.680	-0.691
**I_{corr}(×10^{-3}) / A cm-2**	1.53	2.61	5.68	2.07	6.87

Table S3. Electrochemical impedance parameters obtained by fitting the Nyquist plots of NiCoFe-LDH, RuO_2, and NF to the equivalent circuit mode.

**Catalysts**	R_s	CPE1-T	CPE1-P	R_{ct}
**NiCoFe-LDH**	1.2344	1.5260	0.6207	0.4704
**RuO_2**	1.2137	0.0242	0.6414	8.2383
**NF**	1.1997	0.0069	0.9072	90.5950

Table S4. A comparison of the electrochemical SOR activities of this work with recent outstanding reported electrocatalysts.

Catalyst	Condition	E / V vs.RHE @10 mA cm^{-2}	Reference
**NiCoFe-LDH**	**0.1 M Na_2S+1 M KOH**	**0.381 V**	**This work**
HEDP-Rh	4 M Na_2S + 1 M KOH	0.385 V	[11]
Co-Ni_3S_2	1 M Na_2S + 1 M NaOH	0.39 V	[12]
NiS_2	0.05 M Na_2S (pH=13)	0.41 V	[13]
WS_2 NSs	1 M Na_2S + 1 M NaOH	0.48 V	[14]
PdCo/C	1 M Na_2S + 3 M NaOH	0.69 V	[15]
TiO_2/GC	1 M Na_2S + 3 M NaOH	0.77 V	[16]
PdIr/C	1 M Na_2S + 3 M NaOH	0.85 V	[17]
CoS	1 M Na_2S + 3 M NaOH	0.90 V	[18]

References

1. Yu, Z.; Boukhvalov, D.W.; Tan, H.; Xiong, D.; Feng, C.; Wang, J.; Wang, W.; Zhao, Y.; Xu, K.; Su, W.; et al. Sulfur and phosphorus co-doped FeCoNiCrMn high-entropy alloys as efficient sulfion oxidation reaction catalysts enabling self-powered asymmetric seawater electrolysis. *Chem. Eng. J.* **2024**, *494*, 153094.
2. Zhu, Y.; Wang, S.; Chen, Y.; Zhang, Y.; Feng, Y.; Zhang, G. Screened d-p Orbital Hybridization in Turing Structure of Confined Nickel for Sulfion Oxidation Accelerated Hydrogen Production. *Angew. Chem. Int. Ed.* **2024**, *64*, e202419572.
3. Feng, C.; Lv, M.; Shao, J.; Wu, H.; Zhou, W.; Qi, S.; Deng, C.; Chai, X.; Yang, H.; Hu, Q.; et al. Lattice Strain Engineering of Ni_2P Enables Efficient Catalytic Hydrazine Oxidation-Assisted Hydrogen Production. *Adv. Mater.* **2023**, *35*, 2305598.
4. Huang, J.-L.; Zhang, H.; Suo, T.-Y.; Cunha, J.; Yu, Z.-P.; Xu, W.-Y.; Chen, L.; Hou, Z.-H.; Yin, H. Ru nanoclusters immobilized in N-doped porous carbon for efficient hydrazine-assisted hydrogen production and Zn–hydrazine battery. *Rare Metals* **2025**, *44*, 2502-2512.
5. Pei, A.; Xie, R.K.; Zhu, L.H.; Wu, F.S.; Huang, Z.A.; Pang, Y.Y.; Chang, Y.C.; Chai, G.L.; Pao, C.W.; Gao, Q.S.; et al. Methanol-Enhanced Low-Cell-Voltage Hydrogen Generation at Industrial-Grade Current Density by Triadic Active Sites of Pt_1-Pd_n-(Ni,Co)$(OH)_x$. *J. Am. Chem. Soc.* **2025**, *147*, 3185-3194.
6. Xu, K.; Liang, L.; Li, T.; Bao, M.; Yu, Z.; Wang, J.; Thalluri, S.M.; Lin, F.; Liu, Q.; Cui, Z.; et al. $Pt_{1.8}Pd_{0.2}CuGa$ Intermetallic Nanocatalysts with Enhanced Methanol Oxidation Performance for Efficient Hybrid Seawater Electrolysis. *Adv. Mater.* **2024**, *36*, 2403792.
7. Liu, W.-J.; Xu, Z.; Zhao, D.; Pan, X.-Q.; Li, H.-C.; Hu, X.; Fan, Z.-Y.; Wang, W.-K.; Zhao, G.-H.; Jin, S.; et al. Efficient electrochemical production of glucaric acid and H_2 via glucose electrolysis. *Nat. Commun.* **2020**, *11*, 265.
8. Shi, H.; Wang, T.Y.; Lin, Z.J.; Liu, S.X.; Liu, X.; Zhou, R.X.; Cai, Z.; Huang, Y.H.; Li, Q. Spontaneous Hydrogen Production Coupled with Glucose Valorization through Modulating Au-Pt Coordination on Ultrathin Au_3Pt Twin Nanowires. *Angew. Chem., Int. Ed.* **2025**, *64*, e202424476.
9. Huang, C.-J.; Xu, H.-M.; Shuai, T.-Y.; Zhan, Q.-N.; Zhang, Z.-J.; Li, G.-R. Modulation Strategies for the Preparation of High-Performance Catalysts for Urea Oxidation Reaction and Their Applications. *Small* **2023**, *19*, 2301130.
10. Wang, T.; Miao, L.; Zheng, S.; Qin, H.; Cao, X.; Yang, L.; Jiao, L. Interfacial Engineering of Ni_3N/Mo_2N Heterojunctions for Urea-Assisted Hydrogen Evolution Reaction. *ACS Catal.* **2023**, *13*, 4091-4100.
11. Wang, Z.; Yang, G.; Tian, P.; Li, X.; Deng, K.; Yu, H.; Xu, Y.; Wang, H.; Wang, L. Hydrophilic functionalization of rhodium metallene for saving-energy hydrogen production and sulfur recovery. *Chem. Eng. J.* **2023**, *473*, 145147.
12. Yufei, L.; Yuanxiao, D.; Kai, Z.; Wenzheng, Y. Efficient anodic chemical conversion to boost hydrogen evolution with low energy consumption over cobalt-doped nickel sulfide electrocatalyst. *Chem. Eng. J.* **2022**, *433*, 134472.
13. Zhang, S.; Zhou, Q.; Shen, Z.; Jin, X.; Zhang, Y.; Shi, M.; Zhou, J.; Liu, J.; Lu, Z.; Zhou, Y.N.; et al. Sulfophobic and Vacancy Design Enables Self-Cleaning Electrodes for Efficient Desulfurization and Concurrent Hydrogen Evolution with Low Energy Consumption. *Adv. Funct. Mater.* **2021**, *31*, 2101922.
14. Yi, L.; Ji, Y.; Shao, P.; Chen, J.; Li, J.; Li, H.; Chen, K.; Peng, X.; Wen, Z. Scalable Synthesis of Tungsten Disulfide Nanosheets for Alkali-Acid Electrocatalytic Sulfion Recycling and H_2 Generation. *Angew. Chem. Int. Ed.* **2021**, *60*, 21550-21557.
15. Kim, K.; Han, J.-I. Carbon supported bimetallic Pd–Co catalysts for alkaline sulfide oxidation in direct alkaline sulfide fuel cell. *Int. J. Hydrogen Energy* **2015**, *40*, 4567-4572.

16. Sahoo, P.C.; Kim, K.; Lee, J.H.; Han, J.-I.; Oh, Y.-K. Biomimetically Synthesized Hierarchical TiO_2-Graphitic Carbon as Anodic Catalysts for Direct Alkaline Sulfide Fuel Cell. *ACS Sustain. Chem. Eng.* **2015**, *3*, 1764-1770.
17. Kim, K.; Han, J.-I. Carbon-supported bimetallic Pd–Ir catalysts for alkaline sulfide oxidation in direct alkaline sulfide fuel cell. *J. Appl. Electrochem.* **2015**, *45*, 533-539.
18. Kim, K.; Son, J.; Han, J.-I. Metal sulfides as anode catalysts in direct alkaline sulfide fuel cell. *Int. J. Hydrogen Energy* **2014**, *39*, 10493-10497.
